# Beyond weight loss: exploring bile acid modulations after bariatric surgery and their impact on type 2 diabetes across 5 years

**DOI:** 10.1002/oby.24308

**Published:** 2025-06-15

**Authors:** John Zhiyong Yang, Michelle Li, Weiyu Zhou, Reza Nemati, Xiaodong Jin, Lindsay D. Plank, Rinki Murphy, Jun Lu

**Affiliations:** ^1^ Quzhou Eco‐Industrial Innovation Institute Zhejiang University of Technology Hangzhou Zhejiang China; ^2^ Department of Food and Agriculture Technology Yangtze Delta Region Institute of Tsinghua University Jiaxing China; ^3^ Auckland Bioengineering Institute University of Auckland Auckland New Zealand; ^4^ Business School University of Auckland Auckland New Zealand; ^5^ Canterbury Health Laboratories Canterbury District Health Board Christchurch New Zealand; ^6^ The First Affiliated Hospital of Zhejiang Chinese Medical University (Zhejiang Provincial Hospital of Chinese Medicine) Hangzhou China; ^7^ Department of Surgery, Faculty of Medical and Health Sciences University of Auckland Auckland New Zealand; ^8^ Department of Medicine, Faculty of Medical and Health Sciences University of Auckland Auckland New Zealand; ^9^ Department of Diabetes Te Toka Tumai, Te Whatu Ora Auckland New Zealand; ^10^ Specialist Weight Management Service Te Mana Ki Tua, Te Whatu Ora Counties South Auckland New Zealand; ^11^ Maurice Wilkins Centre for Biodiscovery Auckland New Zealand; ^12^ School of Chemical Sciences Faculty of Science, University of Auckland Auckland New Zealand

## Abstract

**Objective:**

This study aimed to explore the dynamic role of bile acids (BAs) in metabolic improvements following bariatric surgery, specifically comparing the effects of silastic ring laparoscopic Roux‐en‐Y gastric bypass (SR‐LRYGB) and laparoscopic sleeve gastrectomy (LSG) on BA composition and clinical parameters over a 5‐year period.

**Methods:**

A cohort of patients with obesity and type 2 diabetes underwent SR‐LRYGB or LSG. Principal component analysis was performed to evaluate BAs and clinical outcomes.

**Results:**

Despite significant increases in the first year after surgery, BA levels returned to baseline after 5 years. However, principal component analysis revealed that certain BA profiles may contribute to long‐term metabolic benefits.

**Conclusions:**

Although total BA levels return to baseline by 5 years after surgery, specific BA profiles, as identified by principal component analysis from oral glucose tolerance test data, are associated with sustained metabolic improvements. SR‐LRYGB is associated with more durable metabolic benefits compared with LSG. However, given the use of the oral glucose tolerance test, which may not fully capture postprandial BA dynamics, further research using mixed‐meal tolerance tests is needed to confirm these findings.

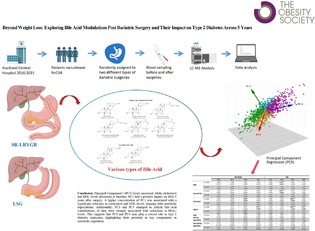

## Graphical abstract

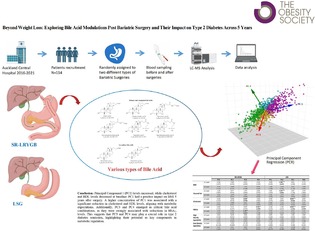

**Study Importance**

**What is already known?**

- Bariatric surgery significantly alters gastrointestinal anatomy, leading to changes in hormonal and metabolic pathways.
- As signaling molecules, bile acids (BAs) influence glucose and lipid metabolism, energy expenditure, and insulin sensitivity. Previous studies have shown that bariatric surgery‐induced changes in BA composition and concentration are associated with improvements in glucose metabolism.

**What does this study add?**

- Despite their significant changes in the first year after surgery, BA levels returned to baseline after 5 years.
- Higher principal component 1 (PC1) levels were associated with a significant reduction in cholesterol and high‐density lipoprotein. PC3 and PC4 emerged as BA combinations associated with reductions in hemoglobin A1c, which suggests that they may play a crucial role in type 2 diabetes remission.

**How might these results change the direction of research or the focus of clinical practice?**

- Findings emphasize the need for ongoing monitoring and individualized management of patients after bariatric surgery to sustain metabolic benefits.
- Results call for further investigation into alternative mechanisms that contribute to long‐term metabolic improvements after bariatric surgery. Although BAs may have a role in the short term, other factors are likely more critical for maintaining glycemic control and weight regulation over time.

## INTRODUCTION

Obesity represents a burgeoning global health crisis, driving the epidemic of type 2 diabetes (T2D) [1, 2]. The surge in obesity‐related metabolic disorders has fueled an urgent quest for interventions that extend beyond mere weight reduction to address the metabolic dysregulations accompanying these conditions. Among such strategies, bariatric surgeries, such as laparoscopic sleeve gastrectomy (LSG) and silastic ring laparoscopic Roux‐en‐Y gastric bypass (SR‐LRYGB), demonstrate profound metabolic effects related to the weight loss achieved [3, 4].

The multifaceted nature of different types of bariatric surgeries has propelled an exploration into their underlying mechanisms of action, uncovering a dynamic interplay between anatomical alterations, hormonal modifications, potential gut–brain mechanisms, and metabolic rearrangements [5, 6, 7]. Of particular interest is the potential role played by bile acids (BAs), bioactive molecules at the nexus of lipid metabolism, glucose homeostasis, and energy regulation, which are thought to orchestrate favorable metabolic changes after surgery [8].

BAs, traditionally known for their role in lipid emulsification and absorption, have emerged as signaling molecules intricately linked to metabolic regulation. Studies have illuminated their diverse functions, encompassing modulation of cellular signaling pathways, regulation of energy expenditure, and influence on glucose and lipid metabolism via activation of specific nuclear receptors and gut hormone secretion [9, 10, 11]. Specifically, the farnesoid X nuclear receptor (FXR), G‐protein‐coupled BA receptor 1 (TGR5), fibroblast growth factor 19 (FGF19), and glucagon‐like peptide‐1 (GLP‐1) have been extensively studied and shown to correlate with changes in BA levels [12, 13, 14, 15]. Importantly, alterations in BA composition have been implicated in the pathophysiology of T2D, underscoring their potential as therapeutic targets in metabolic diseases [8, 16].

The transformative impact of LSG and SR‐LRYGB surgeries on metabolic health goes beyond weight loss, heralding rapid and sustained improvements in glucose homeostasis, insulin sensitivity, and T2D remission [17, 18]. Concomitant with these metabolic shifts, these procedures induce intricate alterations in the enterohepatic circulation of BAs, prompting a deeper investigation into the dynamic changes in BA profiles and their potential causal contributions to metabolic outcomes [19, 20]. Studies have also demonstrated a relationship between significant improvements in glycemic response and alterations in BA profiles and signaling in patients with severe obesity following bariatric surgery [21]. Overall, a significant increasing trend in primary and secondary BA levels was observed 12 months after SR‐LRYGB, but no change was found in LSG. Both conjugated and unconjugated BA levels increased in patients after SR‐LRYGB, whereas they did not change significantly in patients following LSG [22].

This article aims to investigate the temporal BA changes following SR‐LRYGB and LSG surgeries over a 5‐year period and their correlations with various clinical parameters and the remission trend of T2D.

## METHODS

### Study design

The study was a randomized, double‐blind trial with two parallel arms, aiming to assess the following: 1) the relationship between various types of temporal BAs and specific clinical parameters; and 2) the relative effectiveness of two laparoscopic procedures, SR‐LRYGB and LSG, in achieving remission of T2D in relation to these BAs' changes. The surgeries were performed at a single hospital center in Auckland, New Zealand. All the participants provided written informed consent. The study was approved by the Northern A Health and Disability Ethics Committee of the Ministry of Health, New Zealand (NZ93405). The trial design protocol was published in detail [23].

### Participant recruitment

The study recruited individuals aged between 20 and 55 years old with T2D for a minimum duration of 6 months, with a body mass index (BMI) of 35 to 65 kg/m^2^ for at least 5 years. In total, 221 eligible participants consented to undergo either surgical procedure or committed to regular follow‐up, and 114 patients were finally randomized. Detailed information, including a CONSORT diagram, has been published previously [24].

### Procedures

#### Surgical protocol

Before surgery, participants followed a very low‐calorie diet with three OPTIFAST (Nestlé Health Science) servings of around 150 calories and vegetables for 2 weeks. Details of the bariatric surgical procedures were published in detail previously [24].

#### Data collection

In this study, blood samples were collected in the fasted state and at five time points following the 75‐g oral glucose tolerance test (OGTT) over the study period. The data collected before the presurgery stage serve as the baseline for this study. A detailed description of the data collection of the trial was published previously [24].

Beyond glycemic and weight‐related outcomes, additional measures included blood pressure, lipid levels, and BA concentration. The measurement of all individual BAs was conducted using the liquid chromatography–tandem mass spectrometry (LC‐MS/MS) method, as previously described [25]. Area under the curve (AUC) analyses of the indices over 120 minutes (AUC_0‐120min_) are the basis of the study's data collections. The analyzed BAs included chenodeoxycholic acid (CDCA), cholic acid (CA), deoxycholic acid (DCA), lithocholic acid (LCA), ursodeoxycholic acid (UDCA), and their respective glycine (including GUDCA, GCA, GCDCA, and GDCA) and taurine (including TUDCA, TCDCA, TDCA, and TLCA) conjugates.

BA composition was categorized based on synthesis site (primary, including CA, GCA, CDCA, GCDCA, and TCDCA, or secondary, including DCA, GDCA, TDCA, UDCA, GUDCA, TUDCA, LCA, and TLCA), conjugation status (conjugated, including TUDCA, TCDCA, TDCA, and TLCA, or unconjugated, including UDCA, CA, CDCA, DCA, and LCA), and 12α‐hydroxylation (12α‐OH, including CA, GCA, DCA, GDCA, and TDCA, or non–12α‐OH, including CDCA, GCDCA, TCDCA, LCA, TLCA, UDCA, GUDCA, and TUDCA). The total concentration of BAs in each category was determined by summing the molar concentrations of individual BAs.

#### Follow‐up

Postoperative care and follow‐up procedures were standardized for both study groups. From the time of surgery, all pharmacological agents for diabetes were discontinued. However, if the mean postoperative capillary glucose level exceeded 12 mmol/L, glucose‐lowering therapy was reintroduced. To ensure consistent and unbiased management, all patients underwent reviews by endocrinologists for the adjustment of metabolic medications. This adjustment followed a predetermined protocol, and the endocrinologists were blinded to the specific surgical procedure each patient underwent [23, 26].

### Calculation and statistical analysis

The data analysis employed Stata version 18 (StataCorp LLC). The trial was meticulously orchestrated with the primary objective of attaining 80% statistical power to discern the efficacy of two distinct surgical interventions. To accommodate an anticipated 20% loss to follow‐up, a minimum enrollment of 53 patients per group was deemed imperative. Continuous variables conforming to a normal distribution were articulated using means (SD), whereas discrete variables, subject to non‐normal distributions, were characterized using medians and interquartile ranges. Student *t* tests were employed for normally distributed data.

The assessment of the disparity in diabetes remission rates between the SR‐LRYGB and LSG groups at 1‐ and 5‐year intervals involved adjusting for stratification variables using logistic regression. Intragroup alterations and intergroup variances were elucidated through point estimates and 95% confidence intervals (CI).

Correlative analyses linking various BA concentrations with T2D remission were conducted using principal component (PC) regression [27]. AUC calculations, executed according to the trapezoidal rule, contributed to a nuanced understanding of these associations. Statistical significance was defined at *p* < 0.05 (two‐tailed). For hemoglobin A1c (HbA1c) and BMI assessments conducted at baseline and across multiple instances over 1‐ and 5‐year intervals, a repeated‐measures mixed‐effects model was applied.

## RESULTS

### Baseline characteristics of patients

Baseline characteristics of 114 randomized individuals are summarized in Table 1. The mean age was 46.0 (SD 6.6) years, the mean BMI was 42.8 (SD 6.5) kg/m^2^, and the mean HbA1c was 7.9% (SD 1.4%; mean 63.4 [SD 15.6] mmol/mol). Of the 114 participants, 28%, or 32 participants, had T2D for more than 10 years, and 30%, or 34 participants, were treated with insulin. Two participants died during the 5‐year follow‐up period, and four of them lost contact. At 5 years, we obtained the diabetes status of 108 participants (95%), but only 99 participants (87%) attended the other scheduled clinical assessments.

**TABLE 1:** Baseline characteristics of patients.

	SR‐LRYGB (*n* = 56)	LSG (*n* = 58)
Age, mean (SD), y	46.6 (6.7)	45.5 (6.4)
Female, *n* (%)	33 (59)	26 (45)
Ethnicity, *n* (%)		
New Zealand European	34 (61)	38 (66)
Māori/Pacific	17 (31)	13 (23)
Others	5 (9)	7 (12)
Duration of diabetes, *n* (%), y		
<5	26 (46)	24 (41)
5–10	13 (23)	19 (33)
>10	17 (30)	15 (26)
Use of insulin, *n* (%)	17 (30)	16 (28)
HbA1c, mean (SD)		
mmol/mol	64.5 (18.1)	61.9 (12.8)
%	8.1 (1.7)	7.8 (1.2)
Body weight, mean (SD), kg	123.4 (21.3)	126.7 (24.5)
BMI, *n* (%), kg/m^2^		
35–44.9	43 (77)	41 (71)
45–54.9	9 (16)	15 (26)
55–65	4 (7)	2 (3)
Lipid profile, mean (SD)		
Total cholesterol, mmol/L	4.46 (0.19)	4.3 (0.23)
Triglycerides, mmol/L	2.06 (0.19)	1.6 (0.15)
HDL, mmol/L	1.05 (0.05)	1.09 (0.04)
LDL, mmol/L	2.47 (0.16)	2.65 (0.20)

Abbreviations: HbA1c, hemoglobin A1c; HDL, high‐density lipoprotein; LDL, low‐density lipoprotein; LSG, laparoscopic sleeve gastrectomy; SR‐LRYGB, silastic ring laparoscopic Roux‐en‐Y gastric bypass.

### Comparison of baseline and postsurgery end points at 1 year and 5 years

Table 2 provides a summary of the changes observed in weight loss, BMI, and HbA1c levels for SR‐LRYGB and LSG surgeries, highlighting the results over various time intervals. Significant weight loss was noted for both procedures, but SR‐LRYGB resulted in superior long‐term weight maintenance [24]. By the 5‐year mark, patients with SR‐LRYGB had a 23% body weight loss compared with 15% for LSG. Excess weight loss was similarly higher for SR‐LRYGB participants at both 1 year (79% vs. 69%) and 5 years (64% vs. 41%). In terms of BMI, both groups experienced reductions, but participants with SR‐LRYGB had more stable BMI levels over time. HbA1c levels significantly decreased in both groups, with SR‐LRYGB participants maintaining lower levels over 5 years, indicating better long‐term diabetes management.

**TABLE 2:** Presurgery and postsurgery 1‐year and 5‐year end point comparison.

	SR‐LRYGB	LSG	*p* value
Body weight, mean (SD), kg			
Presurgery	115.9 (22.2)	118.2 (22.2)	
Postsurgery 1‐y end point	82.8 (716.5)	87.7 (16.3)	
Postsurgery 5‐y end point	89 (18.4)	100 (18)	
Percentage weight loss, mean % (95% CI)			
Presurgery to 1‐y end point	**−28.6 (3.26)** ***	**−25.8 (3.56)** ***	0.22
Presurgery to 5‐y end point	**−23.2 (2.89)** ***	**−15.2 (2.14)** ***	**0.00** ***
1‐y end point to 5‐y end point	7.5 (0.45)	**14.0 (1.32)** ***	**0.00** ***
Excess weight loss, mean % (95% CI)			
Presurgery to 1‐y end point	**−79.2 (15.22)** ***	**−69.2 (12.96)** ***	**0.01** ***
Presurgery to 5‐y end point	**−64.4 (12.22)** ***	**−41.3 (8.04)** ***	**0.00** ***
1‐y end point to 5‐y end point	**71.3 (7.32)** ***	**90.4 (5.88)** ***	**0.00** ***
BMI, mean (SD), kg/m^2^			
Presurgery	39.1 (6.2)	39.9 (6.2)	
Postsurgery 1‐y end point	28.3 (4.7)	30.5 (4.7)	
Postsurgery 5‐y end point	29.9 (5.5)	34.5 (5.4)	
Difference, kg/m^2^ (95% CI)			
Presurgery to 1‐y end point	**−10.8 (3.96)** ***	**−9.4 (9.68)** ***	0.35
Presurgery to 5‐y end point	**−9.2 (12.26)** ***	**−5.4 (8.19)** ***	0.25
1‐y end point to 5‐y end point	1.6 (0.23)	**4.0 (2.89)** **	**0.00** ***
HbA1c (without diabetes medication) <6.0% (42 mmol/mol), *n* (%)			
Presurgery	3 (5.26)	0 (0)	
Postsurgery 1‐y end point	28 (50.88)	32 (55.17)	
Postsurgery 5‐y end point	28 (49.12)	26 (37.93)	
Difference, *n* (95% CI)			
Presurgery to 1‐y end point	**25 (2.14)** ***	**32 (1.95)** ***	0.288
Presurgery to 5‐y end point	**25 (2.89)** ***	**26 (3.26)** ***	0.267
1‐y end point to 5‐y end point	0 (2.88)	**−6 (4.02)** **	**0.05** **

*Note*: Data are presented as *n* (%) unless otherwise specified. *P* values indicate comparisons between the two types of surgeries.

Abbreviations: HbA1c, hemoglobin A1c; LSG, laparoscopic sleeve gastrectomy; SR‐LRYGB, silastic ring laparoscopic Roux‐en‐Y gastric bypass.

** *p* < 0.05.

*** *p* < 0.01.

### Effect of SR‐LRYGB and LSG procedures on various levels of BA composition

**FIGURE 1:**
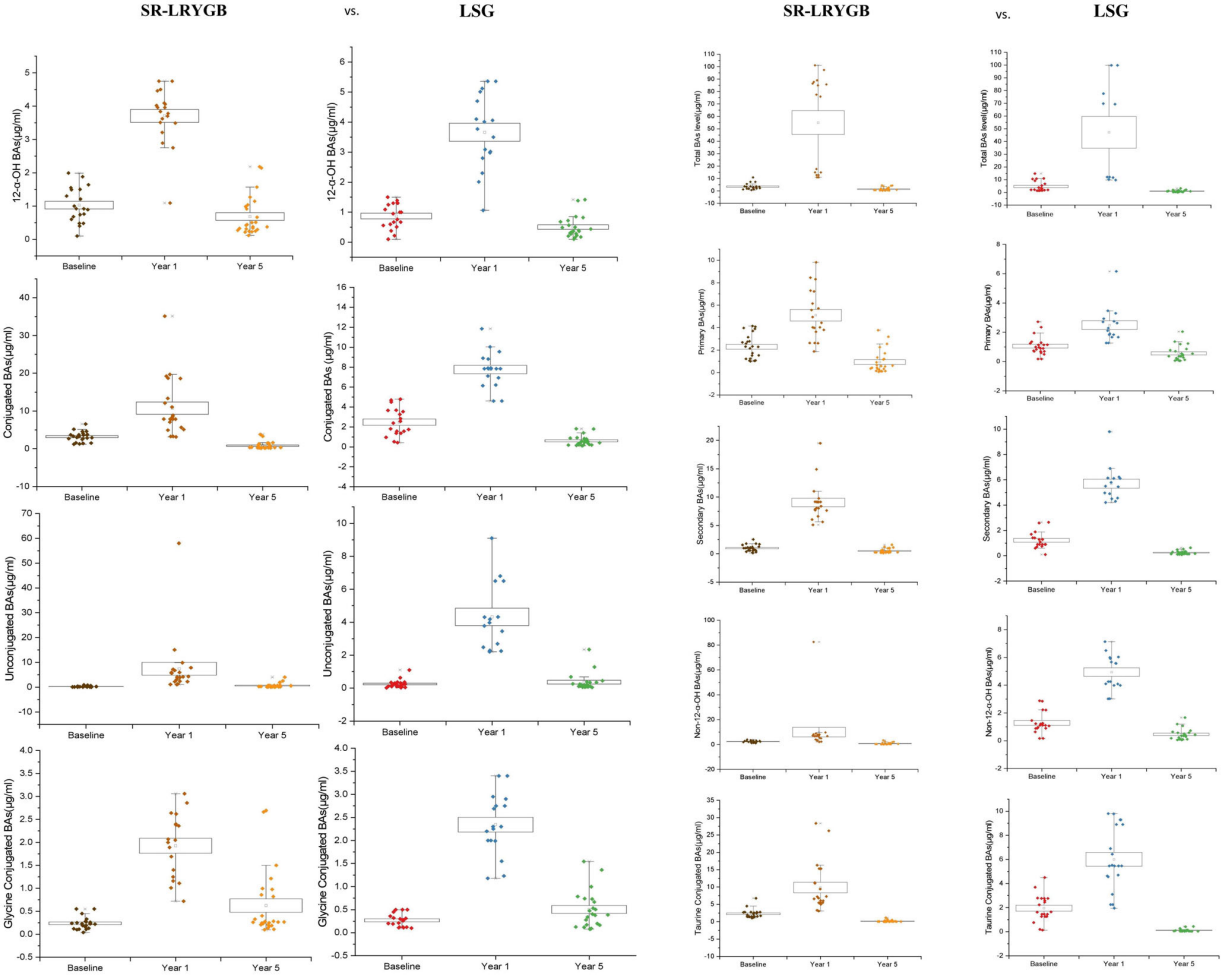
Comparison of various levels of BAs at baseline (presurgery), 1 year, and 5 years after SR‐LRYGB and LSG. The concentration was recorded as micrograms per milliliter. Significant differences (*p* < 0.01) were observed in all groups of BAs between baseline and the 1‐year end point, as well as between the 1‐year and 5‐year end points. BA, bile acid; LSG, laparoscopic sleeve gastrectomy; SR‐LRYGB, silastic ring laparoscopic Roux‐en‐Y gastric bypass. [Color figure can be viewed at wileyonlinelibrary.com]

The changes in various BA levels following SR‐LRYGB compared with LSG at the 1‐ and 5‐year end points exhibited a consistent pattern (Figure 1). For SR‐LRYGB participants, significant differences (*p* < 0.01) were observed in all groups of BA levels, increasing between baseline and the 1‐year end point, then decreasing between the 1‐ and 5‐year end points. Hence, no significant difference was noted in BA levels between baseline and the 5‐year end point. Specifically, the mean total BA levels increased from 4.5 μg/mL at baseline to 55 μg/mL at 1 year after surgery, subsequently decreasing to 0.8 μg/mL at the 5‐year end point. Similarly, primary BAs increased from 2.4 μg/mL at baseline to 5.1 μg/mL at 1 year after surgery, then dropped to 0.5 μg/mL at the 5‐year end point. Secondary BA levels exhibited a similar trend, increasing from 1.3 μg/mL at baseline to 7.9 μg/mL at 1 year after surgery and decreasing to 0.2 μg/mL at the 5‐year end point. Likewise, 12α‐OH BA levels increased from 0.4 μg/mL at baseline to 9.4 μg/mL at 1 year after surgery, then decreased to 0.2 μg/mL at the 5‐year end point. Non–12α‐OH BA, conjugated BA, unconjugated BA, glycine‐conjugated BA, and taurine‐conjugated BA levels followed a similar trend, showing significant increases at the 1‐year end point followed by decreases at the 5‐year end point. Specifically, non–12α‐OH BA levels increased from 0.9 μg/mL at baseline to 3.65 μg/mL at 1 year after surgery but dropped to 0.23 μg/mL at the 5‐year end point. Conjugated BA levels increased from 4.6 μg/mL at baseline to 10.8 μg/mL at 1 year after surgery but dropped to 0.2 μg/mL at the 5‐year end point, whereas unconjugated BA levels increased from 0.1 μg/mL at baseline to 5.6 μg/mL at 1 year after surgery but dropped to 0.1 μg/mL at the 5‐year end point. Glycine‐conjugated BA levels increased from 0.25 μg/mL at baseline to 1.8 μg/mL at 1 year after surgery but dropped to 0.6 μg/mL at the 5‐year end point, and taurine‐conjugated BA levels increased from 2.3 μg/mL at baseline to 9.8 μg/mL at 1 year after surgery but dropped to 0.2 μg/mL at the 5‐year end point.

**FIGURE 2:**
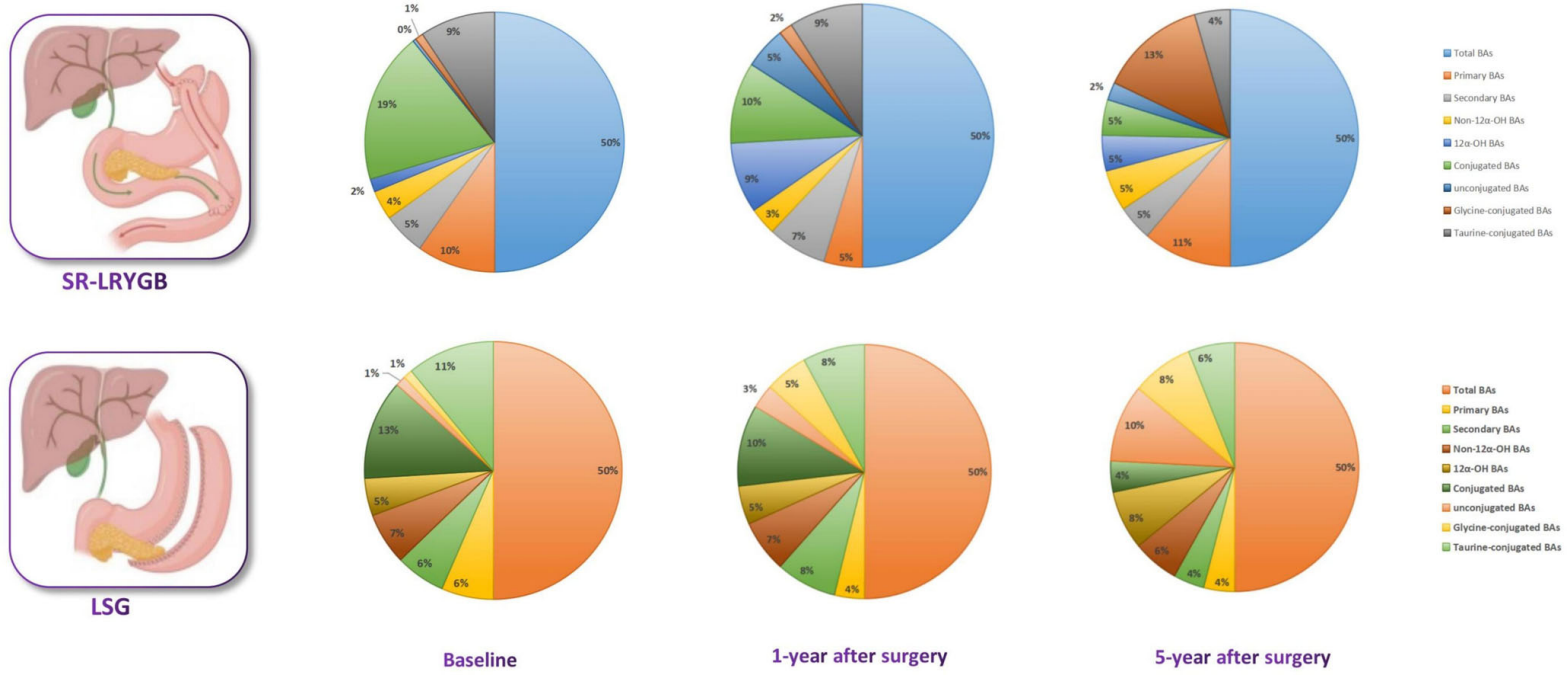
Composition pie charts of the changes in various BAs from baseline (presurgery) to the 1‐year and 5‐year follow‐up points after SR‐LRYGB and LSG procedures. BA, bile acid; LSG, laparoscopic sleeve gastrectomy; SR‐LRYGB, silastic ring laparoscopic Roux‐en‐Y gastric bypass. [Color figure can be viewed at wileyonlinelibrary.com]

Following LSG, changes in various BA levels at both the 1‐ and 5‐year end points exhibited a consistent pattern similar to that of SR‐RYGB, as illustrated in Figure 1. Significant differences (*p* < 0.01) were observed in all groups of BAs, which increased between baseline and the 1‐year end point, then decreased between the 1‐ and 5‐year end points. Consequently, no significant difference was noted between baseline and the 5‐year end point. Notably, the mean total BA levels increased from 4.5 μg/mL at baseline to 46 μg/mL at 1 year after surgery but dropped to 0.3 μg/mL at the 5‐year end point. Primary BA levels increased from 1.25 μg/mL at baseline to 2.8 μg/mL at 1 year after surgery but dropped to 0.2 μg/mL at the 5‐year end point. Similarly, secondary BA levels increased from 1.2 μg/mL at baseline to 5.7 μg/mL at 1 year after surgery but dropped to 0.2 μg/mL at the 5‐year end point. Conjugated BA, unconjugated BA, 12α‐OH BA, non–12α‐OH BA, glycine‐conjugated BA, and taurine‐conjugated BA levels displayed similar trends, showing increases at the 1‐year end point followed by decreases at the 5‐year end point. Specifically, 12α‐OH BA levels increased from 0.9 μg/mL at baseline to 3.6 μg/mL at 1 year after surgery but dropped to 0.38 μg/mL at the 5‐year end point. Non–12α‐OH BA levels increased from 1.25 μg/mL at baseline to 4.9 μg/mL at 1 year after surgery but dropped to 0.3 μg/mL at the 5‐year end point. Conjugated BA levels increased from 2.4 μg/mL at baseline to 7.6 μg/mL at 1 year after surgery but dropped to 0.2 μg/mL at the 5‐year end point. Unconjugated BA levels increased from 0.25 μg/mL at baseline to 2.25 μg/mL at 1 year after surgery but dropped to 0.5 μg/mL at the 5‐year end point. Glycine‐conjugated BA levels increased from 0.22 μg/mL at baseline to 4.02 μg/mL at 1 year after surgery but dropped to 0.4 μg/mL at the 5‐year end point. Taurine‐conjugated BA levels increased from 2.1 μg/mL at baseline to 5.8 μg/mL at 1 year after surgery but dropped to 0.3 μg/mL at the 5‐year end point. Figure 2 provides a summary of the temporal changes in the composition of various BA levels, expressed as percentages for clarity and comparison.

### Correlations between changes in composition of various BAs and HbA1c levels in SR‐LRYGB and LSG procedures

PC analysis was performed to assess the correlation weights of the composition of various BAs. Four key PCs (PC1–PC4) were identified as critical contributors. The results are presented in Table 3.

**TABLE 3:** PC analysis of various BAs.

	PC1	PC2	PC3	PC4
Total BAs	−0.3977	0.1522	0.3694	0.4570
Primary BAs	0.6089	−0.1923	0.2450	−0.0907
Secondary BAs	0.1384	−0.1614	−0.2499	0.5958
Non–12‐α‐OH BAs	0.3470	0.5420	−0.2779	−0.0310
12‐α‐OH BAs	0.4468	0.2375	0.2743	0.2800
Conjugated BAs	0.1983	0.1912	0.5654	−0.0285
Unconjugated BAs	0.2251	−0.3591	−0.2265	0.4881
Glycine‐conjugated BAs	0.0151	−0.5959	0.3605	−0.1292
Taurine‐conjugated BAs	−0.2040	0.2074	0.2960	0.3047

Abbreviations: 12‐α‐OH, 12α‐hydroxylation; BA, bile acid; PC, principal component.

Regression analyses of each PC against clinical parameters—including BMI, visceral fat, cholesterol levels, HbA1c, high‐density lipoprotein (HDL), and low‐density lipoprotein (LDL)—were conducted at baseline, 1 year after surgery, and 5 years after surgery. The findings are summarized in Table 4.

**TABLE 4:** Regression of each clinical variable and PCs at different time points.

	PC1	PC2	PC3	PC4
BMI				
Baseline	−0.191 (0.492)	**0.858** * **(0.517)**	−0.081 (0.545)	−0.428 (0.546)
1 y after surgery	−0.443 (0.366)	**0.805** ** **(0.395)**	**0.705** * **(0.391)**	**0.758** * **(0.424)**
5 y after surgery	**0.963** ** **(0.447)**	0.146 (0.469)	**−1.035** ** **(0.516)**	0.120 (0.544)
Visceral fat				
Baseline	0.028 (0.090)	0.110 (0.096)	−0.034 (0.104)	0.085 (0.101)
1 y after surgery	−0.020 (0.044)	−0.011 (0.048)	0.041 (0.047)	**0.136** *** **(0.051)**
5 y after surgery	0.059 (0.072)	−0.035 (0.076)	−0.017 (0.083)	0.020 (0.088)
Cholesterol				
Baseline	**−0.167** * **(0.085)**	0.026 (0.089)	−0.007 (0.094)	−0.109 (0.094)
1 y after surgery	0.009 (0.073)	0.056 (0.079)	0.072 (0.080)	−0.012 (0.084)
5 y after surgery	−0.012 (0.083)	0.057 (0.086)	−0.114 (0.096)	0.107 (0.100)
HbA1c				
Baseline	−0.115 (0.111)	0.161 (0.116)	**−0.234** * **(0.123)**	0.022 (0.123)
1 y after surgery	−0.122 (0.077)	0.044 (0.083)	0.004 (0.085)	**−0.156** * **(0.089)**
5 y after surgery	0.030 (0.090)	0.064 (0.093)	0.149 (0.103)	0.021 (0.109)
HDL				
Baseline	**−0.039** * **(0.022)**	0.022 (0.023)	−0.030 (0.024)	**−0.053** ** **(0.025)**
1 y after surgery	0.021 (0.027)	−0.026 (0.029)	0.017 (0.030)	−0.029 (0.031)
5 y after surgery	0.011 (0.031)	−0.006 (0.031)	−0.019 (0.035)	−0.004 (0.037)
LDL				
Baseline	−0.081 (0.070)	−0.004 (0.074)	0.050 (0.078)	−0.120 (0.080)
1 y after surgery	−0.006 (0.064)	0.074 (0.070)	0.058 (0.071)	0.002 (0.074)
5 y after surgery	−0.031 (0.071)	0.064 (0.074)	−0.095 (0.082)	0.071 (0.086)

*Note*: Data given as mean (SE).

Abbreviations: HbA1c, hemoglobin A1c; HDL, high‐density lipoprotein; LDL, low‐density lipoprotein; PC, principal component.

* *p* < 0.1.

** *p* < 0.05.

*** *p* < 0.01.

At baseline, BMI was positively correlated with PC2, cholesterol was negatively correlated with PC1, HbA1c was negatively correlated with PC3, and HDL was negatively correlated with PC1 and PC4. One year after surgery, BMI was positively correlated with PC2 to PC4, visceral fat was positively correlated with PC4, and HbA1c was negatively correlated with PC4. Five years after surgery, BMI was positively correlated with PC1 but negatively correlated with PC3, and no significant relationships were observed between any PC groups and other clinical parameters.

General changes between clinical parameters and PCs at 1 and 5 years after surgery were analyzed using regression, with the results presented in Table 5.

**TABLE 5:** Regression of the changes between each clinical variable and PCs at different time points.

	PC1	PC2	PC3	PC4
BMI				
Δ 1‐y	−0.411 (0.719)	**−1.415** * **(0.738)**	0.020 (0.740)	−0.282 (0.821)
Δ 5‐y	−0.782 (0.881)	−0.418 (0.882)	−1.068 (0.867)	−0.233 (0.961)
Visceral fat				
Δ 1‐y	0.091 (1.783)	**−3.381** * **(1.899)**	0.757 (1.830)	0.211 (2.033)
Δ 5‐y	−3.800 (2.531)	−0.825 (2.535)	−2.942 (2.457)	−0.940 (2.782)
Cholesterol				
Δ 1‐y	1.505 (2.384)	−1.936 (2.394)	−0.449 (2.490)	−1.301 (2.528)
Δ 5‐y	−3.283 (2.686)	−0.808 (2.713)	−2.766 (2.742)	3.350 (3.058)
HbA1c				
Δ 1‐y	−1.441 (1.051)	−0.429 (1.054)	**−2.243** ** **(1.096)**	**−3.841** *** **(1.119)**
Δ 5‐y	−0.388 (1.158)	0.056 (1.165)	**−1.979** * **(1.183)**	0.878 (1.289)
HDL				
Δ 1‐y	2.325 (2.405)	0.294 (2.416)	2.285 (2.513)	−1.900 (2.551)
Δ 5‐y	−0.773 (2.454)	−0.478 (2.479)	−0.074 (2.506)	1.095 (2.794)
LDL				
Δ 1‐y	−3.449 (5.862)	−6.410 (5.932)	**4.496** * **(6.271)**	2.714 (6.224)
Δ 5‐y	2.431 (5.826)	0.192 (5.859)	**11.602** ** **(5.880)**	9.751 (6.547)

*Note*: Data given as mean (SE). Δ 1‐year: change between 1 year after surgery and baseline; Δ 5‐year: change between 5 years after surgery and baseline.

Abbreviations: HbA1c, hemoglobin A1c; HDL, high‐density lipoprotein; LDL, low‐density lipoprotein; PC, principal component.

* *p* < 0.1.

** *p* < 0.05.

*** *p* < 0.01.

For changes between 1 year and baseline, BMI and visceral fat were negatively correlated with PC2, whereas HbA1c was negatively correlated with both PC3 and PC4, and LDL was positively correlated with PC3. Between 5 years and baseline, PC3 was negatively correlated with HbA1c but positively associated with LDL.

The specific changes in clinical parameters and PCs based on the two surgical procedures (SR‐LRYGB and LSG) at 1 and 5 years after surgery were also analyzed using regression, as shown in Table 6.

**TABLE 6:** Regression analysis of changes in clinical variables and PCs at different time points by surgery type.

	SR‐LRYGB				LSG			
	PC1	PC2	PC3	PC4	PC1	PC2	PC3	PC4
BMI								
Δ 1‐y	−0.534 (1.067)	0.588 (1.056)	−0.272 (1.050)	−0.142 (1.243)	−0.409 (0.933)	**−2.946** *** **(1.007)**	0.601 (1.000)	−0.753 (1.045)
Δ 5‐y	−0.522 (1.224)	−0.369 (1.252)	−1.723 (1.338)	0.379 (1.622)	−1.608 (1.330)	−0.629 (1.282)	−0.031 (1.110)	−1.096 (1.103)
Visceral fat								
Δ 1‐y	−1.126 (3.004)	2.077 (3.035)	−1.797 (2.954)	2.246 (3.575)	1.920 (1.653)	**−9.293** *** **(1.897)**	2.907 (1.736)	−2.593 (1.803)
Δ 5‐y	−4.561 (3.533)	−2.974 (3.652)	−3.907 (3.824)	−0.502 (4.652)	−4.561 (3.533)	−2.974 (3.652)	−3.907 (3.824)	−0.502 (4.652)
Cholesterol								
Δ 1‐y	−0.316 (3.787)	−0.592 (3.529)	**−6.405** * **(3.735)**	−4.103 (3.815)	−0.439 (2.672)	0.343 (2.894)	**−8.034** *** **(2.919)**	2.094 (2.910)
Δ 5‐y	0.325 (2.939)	−0.908 (2.961)	−0.421 (3.171)	4.763 (3.857)	−9.986* (5.071)	−0.242 (5.387)	−6.229 (4.825)	2.255 (4.833)
HbA1c								
Δ 1‐y	−0.663 (1.450)	1.312 (1.347)	−0.007 (1.425)	**−3.258** ** **(1.469)**	−2.520 (1.511)	−1.437 (1.637)	**−4.922** *** **(1.651)**	**−4.033** ** **(1.646)**
Δ 5‐y	−0.401 (1.281)	0.389 (1.304)	**−3.160** ** **(1.417)**	2.077 (1.708)	−0.544 (2.240)	−1.117 (2.293)	0.018 (2.056)	−0.361 (1.974)
HDL								
Δ 1‐y	−0.915 (3.705)	0.678 (3.453)	3.916 (3.654)	−6.109 (3.733)	4.079 (3.329)	0.601 (3.605)	1.303 (3.637)	1.169 (3.625)
Δ 5‐y	−2.350 (3.542)	−2.178 (3.569)	−0.009 (3.821)	0.391 (4.649)	2.884 (3.219)	1.483 (3.420)	−0.668 (3.063)	1.517 (3.068)
LDL								
Δ 1‐y	−12.321 (11.014)	−3.781 (10.369)	−15.149 (11.345)	−0.283 (11.096)	−2.438 (4.859)	−3.260 (5.262)	8.774 (5.308)	4.923 (5.291)
Δ 5‐y	6.419 (7.202)	0.841 (7.256)	−8.803 (7.770)	12.952 (9.452)	−6.497 (10.375)	0.123 (10.887)	−15.038 (9.498)	7.547 (9.442)

*Note*: Data given as mean (SE). Δ 1‐year: change between 1 year after surgery and baseline; Δ 5‐year: change between 5 years after surgery and baseline.

Abbreviations: HbA1c, hemoglobin A1c; HDL, high‐density lipoprotein; LDL, low‐density lipoprotein; LSG, laparoscopic sleeve gastrectomy; PC, principal component; SR‐LRYGB, silastic ring laparoscopic Roux‐en‐Y gastric bypass.

* *p* < 0.1.

** *p* < 0.05.

*** *p* < 0.01.

#### SR‐LRYGB

For changes between 1 year and baseline, cholesterol was negatively correlated with PC3, and HbA1c was negatively associated with PC4. Between 5 years and baseline, only HbA1c showed a negative association with PC3.

#### LSG

For changes between 1 year and baseline, BMI and visceral fat were negatively correlated with PC2, cholesterol was negatively associated with PC3, and HbA1c was negatively correlated with PC3 and PC4. After 5 years, no significant relationships were observed.

## DISCUSSION

Our OGTT‐based findings demonstrate that BA levels return to baseline by 5 years after surgery, contrasting with mixed‐meal tolerance test studies reporting prolonged elevation—a divergence that may reflect differences in nutrient‐stimulated enterohepatic signaling, which needs further evaluation [28]. BAs have been widely studied for their role in metabolic improvements after bariatric surgery, although their long‐term contributions remain debated. For example, procedures such as LSG and SR‐LRYGB are associated with transient increases in circulating BA levels postoperatively, including elevated 12α‐OH species linked to short‐term glucose regulation. However, whereas patients with SR‐LRYGB exhibit pronounced rises in total, conjugated, and unconjugated BA levels compared with LSG patients, the relevance of these early surges to sustained metabolic outcomes remains unclear, underscoring the need to interpret BA dynamics within the context of testing methodology and surgical technique [22, 29].

In this study, we found that both types of bariatric surgery, SR‐LRYGB and LSG, had a comparable impact on reducing excess weight, which contributed to a decrease in BMI. Regarding T2D remission, both procedures showed similar outcomes in achieving HbA1c levels within the cutoff criteria 1 year after surgery. However, over a longer period, SR‐LRYGB proved significantly more effective than LSG in sustaining T2D remission at 5 years after surgery. The results matched the previous studies [30].

Because the composition of various BAs changes dynamically over time, it is challenging to use a single data point as an independent variable for statistical analysis (Figure 2). Our study used the PC analysis method to identify the optimal composition of various BAs and conducted regression analyses to examine the relationship between these PCs and the clinical parameters collected. This approach allowed us to effectively explore the association between BAs and clinical outcomes, as well as how changes in BA levels over time correlate with changes in clinical outcomes.

The random overall regression results revealed the following: 1) as PC1 levels increased, cholesterol and HDL levels decreased at baseline, and PC1 had a positive impact on BMI 5 years after surgery; 2) the concentration of PC2 was positively associated with BMI at baseline and 1 year after surgery; 3) an increase in PC3 concentration led to a decrease in HbA1c levels at baseline, an increase in BMI 1 year after surgery, and a decrease in BMI 5 years after surgery; and 4) PC4 concentration was positively correlated with visceral fat, whereas HbA1c levels significantly decreased 1 year after surgery. Additionally, PC4 concentration was positively associated with BMI 1 year after surgery.

The results indicated that the correlations between BAs and BMI are highly variable, primarily influenced by changes in body mass. However, variations in fat composition and distribution across different ethnicities significantly impact metabolic rates, which could explain the challenges in linking BA concentration directly to BMI. A higher concentration of PC1 was associated with a significant reduction in cholesterol and HDL levels, which aligns with metabolic expectations [31]. Additionally, PC3 and PC4 emerged as critical BA combinations because they were strongly associated with reductions in HbA1c levels. This suggests that PC3 and PC4 may play a crucial role in T2D remission, highlighting their potential as key components in metabolic regulation.

The regression analysis of the overall changes in clinical parameters and various BA composition groups after bariatric surgery revealed distinct associations between BA PCs and metabolic outcomes. Notably, an increase in PC2 levels was linked to a reduction in visceral fat storage, highlighting its potential role in improving body fat composition after surgery. Meanwhile, PC3 levels were strongly associated with significant reductions in HbA1c levels both 1 year and 5 years after surgery, indicating its sustained role in glycemic control over time. In contrast, PC4 levels were only associated with HbA1c reductions at the 1‐year mark, suggesting a more transient influence on glucose metabolism.

Interestingly, PC3 was also linked to a significant increase in LDL levels. Although reductions in HbA1c typically signify improved glucose metabolism, the observed increase in LDL does not necessarily contradict this outcome. LDL fluctuations after surgery can be influenced by various factors, such as dietary changes, genetic predispositions, or shifts in lipid metabolism [32]. Previous studies have demonstrated that bariatric procedures such as SR‐LRYGB often lead to improvements in lipid profiles, but temporary or individual increases in LDL have been observed during certain postoperative phases [33].

These findings suggest that PC3 plays a crucial role in the long‐term remission of T2D, as evidenced by its sustained impact on HbA1c levels. In comparison, PC4 appears to contribute to glycemic control only in the short term (1 year after surgery). Overall, PC3 and PC4 emerged as reliable indicators of metabolic improvements, particularly regarding T2D remission, with PC3 being the more consistent predictor over time.

The regression analysis of changes in clinical parameters and various BA composition groups after bariatric surgery, stratified by surgery type, revealed distinct patterns. For SR‐LRYGB, an increase in the total component levels of PC3 was significantly associated with a decrease in cholesterol levels, whereas an increase in PC4 components was significantly linked to reduced HbA1c levels at the 1‐year checkpoint. Furthermore, the shift in PC3 levels maintained a negative correlation with HbA1c levels, even 5 years after surgery. However, no significant correlations were found between BA composition and reductions in BMI or visceral fat, either in the short or long term. This highlights that in SR‐LRYGB, the BA component PC4 primarily contributed to short‐term reductions in HbA1c, whereas PC3 was critical for maintaining long‐term glycemic control [34].

For LSG, the findings showed that an increase in PC2 levels was associated with significant reductions in BMI and visceral fat 1 year after surgery. Meanwhile, increased PC3 levels were linked to reductions in both cholesterol and HbA1c levels, and PC4 also contributed to HbA1c reduction in the short term. However, no significant relationships were observed at the 5‐year checkpoint for any BA composition group in LSG. These results suggest that LSG's impact on BA composition and clinical outcomes may be more transient compared with SR‐LRYGB [28].

The comparison of these two surgical approaches underscores how the surgery type influences both the amount and composition of BAs, which in turn affects clinical outcomes. Specifically, the changes in PC3 components evoked by SR‐LRYGB were more strongly associated with the sustained remission of T2D over the long term. In contrast, LSG showed more pronounced short‐term effects on BMI, visceral fat, cholesterol, and HbA1c reduction but lacked long‐term correlations.

This novel observation—that SR‐LRYGB evokes PC3 changes that support the prolonged remission of T2D—presents a profound insight that could guide future research. Understanding the mechanisms by which BA composition impacts metabolic outcomes might inform the optimization of surgical approaches for obesity and T2D treatment.

Another novel finding of this study was that the BA levels appear to rise significantly 1 year after both SR‐LRYGB and LSG surgeries but then return to baseline levels by 5 years, despite the well‐described maintenance of weight loss and improved glycemic control among participants with obesity and T2D out to 5 years [35, 36, 37]. This pattern suggests that although the concentration of various BAs may initially contribute to the metabolic adjustments observed after bariatric surgery, they may not be the primary factors responsible for the sustained improvements in weight and HbA1c over the long term. If BAs were causally related to these long‐term benefits, they would not be expected to see their levels returning to baseline after the initial postoperative period. However, our results suggested that the change amount of a specific combination of various BA groups, such as PC3, significantly reduced the level of HbA1c over the long term. This points to a more complex interplay of factors in maintaining long‐term metabolic health, indicating that the concentration of various BAs might function more as short‐term mediators rather than direct agents of enduring metabolic improvements [20, 34, 38, 39, 40]. The composition of various BAs combined with other unknown factors may contribute more function as long‐term regulation on glucose control. Our findings also suggest that the transient increase may have initiated or sustained other metabolic pathways that contribute to the long‐term benefits observed. For instance, BAs are known to influence hepatic steatosis and cholesterol regulation, and their early elevation after surgery could have long‐term impacts on these processes.

Although both SR‐LRYGB and LSG surgeries elicited a dynamic response in BAs, the changes were remarkably similar in both magnitude and time course. This suggests that the alterations in BA concentration are a general response to both types of gut rearrangements following bariatric surgery, rather than being specific to the type of surgical intervention [15, 41, 42]. Given the stability of nutritional restriction between 1 and 5 years after each type of bariatric surgery, the reduction in BA levels back to baseline levels suggests the changes in BAs are not simply following the changes in diet.

Several limitations of this study warrant discussion, particularly regarding its implications for BA dynamics and metabolic outcomes following bariatric surgery. First, the relatively small sample size may limit the statistical power and generalizability of the findings to broader populations, especially considering the variability in BA metabolism across different demographic and ethnic groups. A larger, more diverse cohort could provide more robust insights into these associations.

Second, the absence of a control group limits our ability to definitively establish causal relationships between bariatric surgery, changes in BA composition, and metabolic improvements. Although the correlations observed between PC groups (PC2, PC3, and PC4) and clinical parameters such as BMI, visceral fat, cholesterol, and HbA1c are intriguing, the lack of a control group introduces the possibility that these changes may partly result from external factors such as lifestyle modifications or dietary interventions following surgery. Additionally, this study was limited by the use of an OGTT to assess BA changes after metabolic bariatric surgery. Despite this, we observed that changes in BA levels were associated with early metabolic improvements after surgery but not at 5 years. SR‐LRYGB was associated with more durable metabolic benefits compared to LSG. These findings suggest the need for further research using more physiologic tests such as the meal tolerance test to better understand the role of BAs in long‐term metabolic outcomes.

Third, this study primarily evaluated outcomes within a 5‐year postoperative period, focusing on short‐ to medium‐term effects. Although our findings suggest that BA levels return to baseline by 5 years after surgery, it remains unclear whether this stabilization persists over longer durations or if additional shifts in BA composition emerge beyond this time frame. Future studies with extended follow‐up periods are needed to confirm these trends and to better understand the potential role of BAs in long‐term metabolic health and T2D remission.

Finally, although we identified key differences in BA dynamics between SR‐LRYGB and LSG, our analysis did not comprehensively account for potential confounding factors, such as differences in dietary intake, physical activity levels, or genetic predispositions, which could have influenced BA metabolism and clinical outcomes. These factors should be more rigorously controlled in future research to ensure a clearer understanding of the mechanistic links between surgical procedures, BA alterations, and metabolic improvements.

Despite these limitations, the findings offer valuable insights into the dynamic interplay between bariatric surgery, BA composition, and metabolic outcomes. They highlight the importance of further research to optimize surgical approaches and identify the mechanisms underpinning long‐term benefits in obesity and T2D management.

## CONCLUSION

In conclusion, this study highlights the dynamic changes in BA composition following SR‐LRYGB and LSG surgeries and their association with short‐term and long‐term T2D remission. The findings underscore the need for further research to elucidate the mechanistic role of BAs in postsurgical metabolic improvements.

## AUTHOR CONTRIBUTIONS

John Zhiyong Yang was responsible for research design, methodology, and data processing and wrote the original draft of the manuscript. Weiyu Zhou performed the data collection, data analyses, and methodology. Michelle Li edited the manuscript and performed the data validation. Reza Nemati performed the data acquisition, methodology, and validation. Xiaodong Jin performed the validation and revised the manuscript. Lindsay D. Plank was responsible for data analyses, statistics, supervision, and manuscript editing. Jun Lu was responsible for conceptualization, supervision, funding acquisition, resource, and final editing of the manuscript. Rinki Murphy performed conceptualization, research design, supervision, resources, and manuscript revision. All authors contributed to the implementation of the study, data acquisition, and data verification and approved the manuscript for publication.

## CONFLICT OF INTEREST STATEMENT

The authors declared no conflicts of interest.

## CLINICAL TRIAL REGISTRATION

ClinicalTrials.gov identifier NCT01486680.

## Data Availability

All data supporting the findings of this study are available within the article.
